# Cataract research using electronic health records

**DOI:** 10.1186/1471-2415-11-32

**Published:** 2011-11-11

**Authors:** Carol J Waudby, Richard L Berg, James G Linneman, Luke V Rasmussen, Peggy L Peissig, Lin Chen, Catherine A McCarty

**Affiliations:** 1 Center for Human Genetics, Marshfield Clinic Research Foundation, Marshfield, Wisconsin, USA; 2 Biomedical Informatics Research Center, Marshfield Clinic Research Foundation, Marshfield, Wisconsin, USA; 3 Department of Ophthalmology, Marshfield Clinic - Minocqua Center, Minocqua Wisconsin, USA; 4 Essentia Institute of Rural Health, Duluth, Minnesota, USA

**Keywords:** Cataract, prevalence, risk factors, epidemiology, electronic health record

## Abstract

**Background:**

The eMERGE (electronic MEdical Records and Genomics) network, funded by the National Human Genome Research Institute, is a national consortium formed to develop, disseminate, and apply approaches to research that combine DNA biorepositories with electronic health record (EHR) systems for large-scale, high-throughput genetic research. Marshfield Clinic is one of five sites in the eMERGE network and primarily studied: 1) age-related cataract and 2) HDL-cholesterol levels. The purpose of this paper is to describe the approach to electronic evaluation of the epidemiology of cataract using the EHR for a large biobank and to assess previously identified epidemiologic risk factors in cases identified by electronic algorithms.

**Methods:**

Electronic algorithms were used to select individuals with cataracts in the Personalized Medicine Research Project database. These were analyzed for cataract prevalence, age at cataract, and previously identified risk factors.

**Results:**

Cataract diagnoses and surgeries, though not type of cataract, were successfully identified using electronic algorithms. Age specific prevalence of both cataract (22% compared to 17.2%) and cataract surgery (11% compared to 5.1%) were higher when compared to the Eye Diseases Prevalence Research Group. The risk factors of age, gender, diabetes, and steroid use were confirmed.

**Conclusions:**

Using electronic health records can be a viable and efficient tool to identify cataracts for research. However, using retrospective data from this source can be confounded by historical limits on data availability, differences in the utilization of healthcare, and changes in exposures over time.

## Background

When considering diseases that impact public health worldwide, few would outrank cataracts. Cataracts are the leading cause of blindness worldwide [1]. Global Burden of Disease 2004 from the World Health Organization ranks cataracts as fourth in disabling conditions in the world following hearing loss, refractive errors, and depression. It estimates the prevalence of moderate and severe disability due to cataracts to be 53.8 million for all ages worldwide [2].

While cataracts may be congenital or result from a specific trauma, most cataracts are related to aging. As the age demographic shifts upward in the population, the incidence of age-related cataract will also increase. In the United States it is estimated that 17.2% of those age 40 and older have cataracts, and this rate is projected to increase by 50% by the year 2020 [3]. The prevalence of cataract surgery among Americans aged 40-years and older is estimated at 5.1%, and that is likely to increase by almost 60% by the year 2020 [3]. There is also the suggestion that with the predicted ozone depletion, the rate of cortical cataracts will increase above the expected levels, resulting in an even higher prevalence of cataracts by the year 2050 [4]. Learning to prevent or delay cataract formation will be an essential part of addressing the growing public health problem of cataracts.

A necessary part of learning to prevent or delay the formation of cataracts is to understand what contributes to their formation. Environmental factors previously reported as being associated with increased rates of cataract include: chronic steroid use, smoking, sun exposure, diabetes, and elevated body mass index (BMI) [5]. Possible protective factors reported include higher intake of antioxidants, increased physical activity, and certain medications [6].

The electronic MEdical Records and GEnomics (eMERGE) network was formed to develop, disseminate, and apply methods for performing complex genomic analysis utilizing electronic health record (EHR) systems as a resource to determine diseases and therapeutic outcomes. A primary goal of eMERGE is to develop and validate electronic algorithms that accurately and effectively classify patients with respect to specific medical conditions such as cataracts [7]. Ultimately, validated phenotypes will be applied across medical records at many facilities in order to improve the efficiency of medical research [8].

The purpose of this study was to develop, validate, and use electronic algorithms to identify cases of age-related cataracts in a population-based biobank and to evaluate the prevalence of cataracts and previously established clinical risk factors for developing cataracts using those algorithms.

## Methods

This study was designed as a retrospective review of a well-established cohort utilizing data from a comprehensive EHR. All individuals in the cohort provided written informed consent, and the project was reviewed and approved by the Marshfield Clinic's Institutional Review Board.

### Study Population

This study population was comprised of participants within the Personalized Medicine Research Project (PMRP). The PMRP is a geographically defined, population-based biobank with over 20,000 subjects, age 18-years and above, enrolled from the Marshfield Clinic healthcare system in Central Wisconsin [9]. The biobank includes DNA, plasma, and serum samples collected at the time of consent. The written informed consent document allows ongoing access to medical records, thereby enabling a wide range of medical research. Participants complete questionnaires that include information on smoking history, occupation, and diet.

### Data Collection

Initially, Current Procedural Terminology (CPT) codes in the Marshfield Clinic EHR were used to select individuals who had cataract surgery and were age 50+ years at the time of their earliest cataract surgical procedure. Congenital and traumatic type cataracts were excluded. There were 2881 total surgeries indicated electronically among 1740 unique individuals. The charts were all manually abstracted by a research coordinator for eye, type of cataract, severity of cataract, and visual acuity just prior to surgery. They were also verified to rule out congenital or traumatic type cataracts. This resulted in 2811 valid surgeries and 1703 unique individuals. Information from this manual abstraction was used to improve the positive predictive value of the electronic algorithm.

To identify individuals having cataract diagnosis without surgery, International Classification of Diseases, 9th revision (ICD-9) and CPT codes were used. In addition, Natural Language Processing (NLP) and Intelligent Character Recognition (ICR) were used to help determine a cataract diagnosis and to identify type of cataract. Using NLP, text-based documents in the EHR were searched for the mention of cataract and cataract types in order to determine a cataract diagnosis. Handwritten documents stored electronically in the EHR were searched for cataract type and severity using ICR [10]. Excluding congenital and traumatic cataract diagnoses, 3035 individuals were identified with a cataract diagnosis and no surgery on or before the data cut off date of 1-15-2008. Of those identified, 1717 (56.6%) were verified by manual abstraction identifying eye, cataract type, severity, visual acuity, and were verified as not being congenital or traumatic type cataract. This was done to determine the positive predictive value of the selection using codes, NLP, and ICR. Using a cataract definition requiring at least one cataract surgical procedure code with age 50+ years at earliest surgical procedure, **or **two or more inclusion type diagnosis codes with age 50+ years at earliest inclusion type diagnosis code, **or **one inclusion type diagnosis code with age 50+ years at earliest inclusion type diagnosis **and **one or more NLP/ICR hits, a weighted positive predictive value of 95.6% was reached.

Smoking history was queried at enrollment into PMRP with respect to whether participants had ever smoked at least 100 cigarettes, as well as their current smoking status. Many subjects (27%) had stopped smoking by the time of enrollment in PMRP. The study's primary comparison of smoking as a risk factor compared current smokers at the time of enrollment to those who had never smoked at the time of enrollment.

Dietary intake data were gathered retrospectively using the National Cancer Institute's Dietary History Questionnaire (DHQ) [11] sent to participants after the time of enrollment [12]. The DHQ is comprised of 124 separate food items and asks about portion sizes for most foods. In addition, there are ten questions about nutrient supplement intake. Software from the National Institutes of Health was used for the nutrient analyses of the DHQ data [13]. Analyses for this study focused on the combined intake of antioxidants (vitamins A, E, and C, beta carotene, zinc, selenium, lutein, and lycopene), including intake from supplements. Intake was observed to be highly variable for the individual antioxidants. In order to obtain a single antioxidant score, the individual intakes were first converted to normal scores [14,15] based on the ranking across PMRP subjects, and a mean of the scores for all antioxidants was calculated for each subject.

Baseline high-density lipoprotein (HDL) cholesterol levels were estimated from laboratory results in the EHR. Details of how baseline HDL was determined can be found elsewhere [16], but in brief, this was accomplished by subsetting HDL values to outpatient results prior to use of statins, fibrates, niacin, hormone replacement therapy, and prior to any diagnosis of cancer, diabetes, or hypothyroidism. Further adjustments were made based on the observed population trends in age and BMI.

After screening procedures to eliminate gross errors in height and weight measurements, BMI was estimated from the EHR. The BMI results prior to cataract were preferentially selected when available. Median BMI was calculated for each subject and used in analyses.

Statin use was determined by selecting the earliest date that statin use was mentioned in the EHR. To determine whether steroid medications had been used, diagnoses where treatment was expected to include the use of steroid medications were identified from the EHR. These diagnoses were categorized as to whether suspicion of adrenal steroid use was > 50% or ≤ 50%. For diagnoses where suspicion of adrenal steroid use was > 50%, two or more unique diagnosis dates were required. For diagnoses where suspicion of adrenal steroid use was ≤ 50%, two or more unique diagnosis dates and two or more unique adrenal steroid medication mention dates were required.

### Statistical analysis

Two primary outcome measures were analyzed: 1) the current prevalence of cataract by age; and 2) age at *first *clinical evidence of cataract. Although nearly all subjects have two eyes in which cataracts may develop, it was assumed that many factors affecting both exposures and diagnosis sensitivity could change after a subject's first cataract event, and therefore, the analysis of subsequent cataract events would require a separate evaluation that will not be considered here. Even studies with prospective follow-up often limit analysis to the worst eye, which would generally be the first eye diagnosed and/or operated on, as used in these analyses.

In processing prior to cataract assessment, EHR data for subjects showing any cataract exclusion codes (e.g., traumatic cataract) were right-censored, and this censoring was applied one year prior to the date of their first exclusion code to allow for delayed documentation of the excluding event. Subjects who did not meet the cataract case event definition provided varying periods of observation time. In time-to-event analyses of age at first cataract, such subjects were considered to be at-risk for developing cataract up to their earliest age at either of the following: a) the end of their "observation time" in the EHR; or b) the occurrence of a censoring event. Subjects have medical visits with varying frequency, and it is possible that subjects not seen regularly in the Marshfield Clinic system may have had a cataract that is undocumented in the EHR. For this reason, and based on review of observed visit histories, the final "observation time" for subjects without cataract was defined as the date of the last diagnosis in a year where some diagnoses were also recorded in one or more of the previous four years. Censoring events included cataract exclusion codes and valid cataract codes (including NLP indications) for subjects with such codes who did not meet the event definition.

The simple prevalence of age-related cataract at enrollment in PMRP was summarized by age group with 95% confidence limits. In analyses of potential risk factors, cataract prevalence was defined at the EHR data acquisition (end of December 2007). These analyses used logistic regression models, stratified by gender and adjusted for age (with age covariates based on restricted cubic splines) [15]. Results are summarized with estimates of odds ratios, together with p-values and confidence limits from asymptotic Wald tests. Results for continuous factors (BMI, HDL, and antioxidant intake) are presented for subjects divided into three equal sized groups (lowest, middle, highest). Relative risks were assumed to change to some degree with age, so models included interactions with age, and estimates are provided for ages 40 and 70. Graphical smoothing with cubic splines was used to illustrate age trends in prevalence.

Basic analyses of age at first cataract included Kaplan-Meier estimates, and both log-rank and Wilcoxon tests for differences are reported. The Wilcoxon test is weighted by the number of subjects at risk and is therefore more sensitive to differences at younger ages relative to the log-rank test. Risk factors for age at first cataract were analyzed with proportional hazards regression models, with stratification by birth cohort and with gender as a covariate. Results are summarized with estimates of hazards ratios, together with p-values and confidence limits from asymptotic Wald tests. Hazard ratios were assumed to differ to some degree by birth cohort, so models included interactions with birth cohort, and estimates are provided for the youngest (born 1960 and later) and oldest (born prior to 1940) cohorts. Results are deemed statistically significant at the 5% level (p < 0.05).

## Results

**Figure 1:**
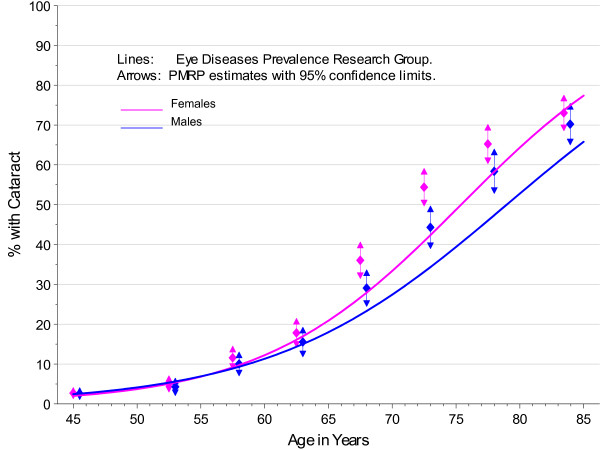
**Prevalence of Cataract by Gender in PMRP and the EDPRG**.

**Figure 2:**
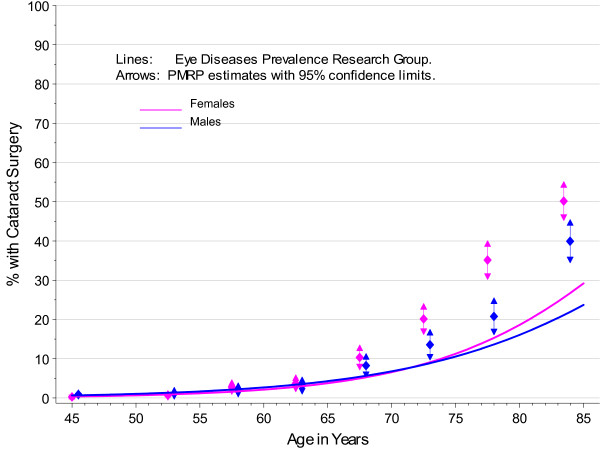
**Prevalence of Cataract Surgery by Gender in PMRP and the EDPRG**.

The PMRP analysis cohort included 19,622 subjects, ages 18 to 98 years (median 46.7 years) at enrollment. Fifty-seven percent (11,222/19,622) were female and 97% were white, non-Hispanic by self-report. The observed prevalence of age-related cataract by age at enrollment in PMRP is shown by gender in Figure 1, together with prevalence estimates for the white U.S. population in year 2000 from the Eye Diseases Prevalence Research Group (EDPRG) [3]. Similarly, the observed prevalence of cataract surgery by age at enrollment in PMRP is shown by gender in Figure 2, together with the EDPRG estimates for pseudophakia/aphakia. The prevalence of age-related cataract below age 30 was extremely low (< 0.2%), and all subsequent analyses were limited to 16,336 PMRP subjects ages 30 and above at the time of data collection (12/31/2007). Table 1 summarizes the characteristics of this analysis cohort.

**Table 1:** Descriptive characteristics of the cataract analysis cohort

	Males	Females	Overall
Subjects (n %)	7,031 43%	9,305 57%	16,336
Cataracts (n %)	1437 20%	2167 23%	3604 22%
Median age of onset (yr)	67.1	65.6	66.2
Minimum	19	13	13
Maximum	90	90	90
Type^1 ^(%)			
Nuclear	96.8%	97.1%	97.0%
Cortical	65.6%	72.1%	69.5%
Posterior subcapsular cataract (PSC)	33.5%	35.9%	34.9%
Cataract surgery (n %)	681 10%	1118 12%	1799 11%
Median age (yr)	56.0	54.9	55.4
Minimum	30	30	30
Maximum	99	98	99
Diabetes (n %)	1243 18%	1305 14%	2548 16%
Smoking history (n %)			
Never	3061 44%	5518 59%	8579 53%
Current	1255 18%	1427 15%	2682 16%
Other or unknown	2715 39%	2360 25%	5075 31%
Steroid use (n %)	1099 16%	1545 17%	2644 16%
Statin use (n %)	2718 39%	2684 29%	5402 33%
Median BMI (kg/m^2^)	28.8	28.0	28.4
Minimum	17.6	15.1	15.1
Maximum	60.1	74.9	74.9
Median adjusted HDL (mg/dL)^2^	46.1	58.4	52.6
Minimum	19.8	22.7	19.8
Maximum	112.0	118.3	118.3
Deceased (n %)	456 6%	376 4%	832 5%

^1^Cataract type available for 2719 subjects with cataract (1610 female, 1109 male.) Subjects may exhibit multiple types.

^2 ^High density lipoprotein (HDL) results were limited to those prior to treatment with statins and prior to diagnosis with a condition known to affect HDL (e.g. thyroid disorders), and were subsequently adjusted for age and body size (body mass index =BMI). At least 2 results were required, and these were available for 7733 subjects (47%).

**Figure 3:**
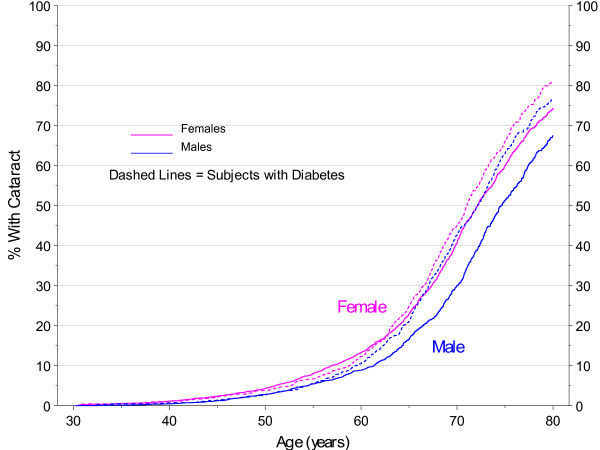
**Cataract Incidence by Age, Kaplan-Meier Estimates by Gender and Diabetes**.

**Figure 4:**
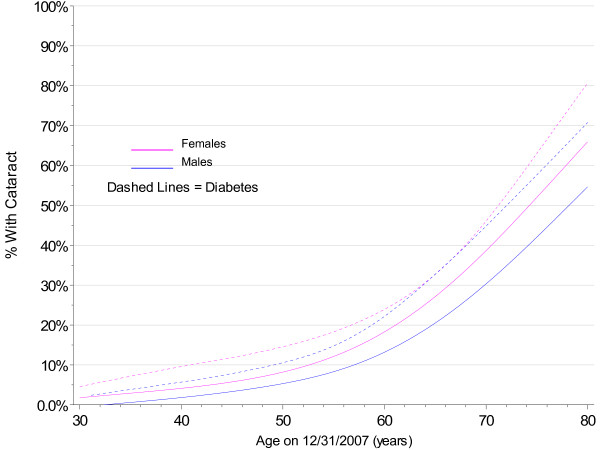
**Smoothed 2007 Prevalence of Cataract by Gender and Diabetes**.

As shown in Figure 3, there were clear differences in age at first cataract by gender (p < 0.0001), with a difference of 2 years in the median age (median 71.7 years in females; 73.7 years in males). There were also differences among those with and without clinical indications of diabetes, but the differences were much stronger in males (both log-rank and Wilcoxon p < 0.0001) than in females (log-rank p = 0.004, Wilcoxon p = 0.498). This is also reflected in Figure 4. To avoid confounding, subsequent analyses of risk factors for cataract were stratified by gender. In addition, clinical guidelines at Marshfield Clinic recommend annual dilated eye exams for patients with diabetes. Since less than 16% of the cohort show clinical indications for diabetes, analyses of other potential risk factors were restricted to those with no indication of diabetes.

**Figure 5:**
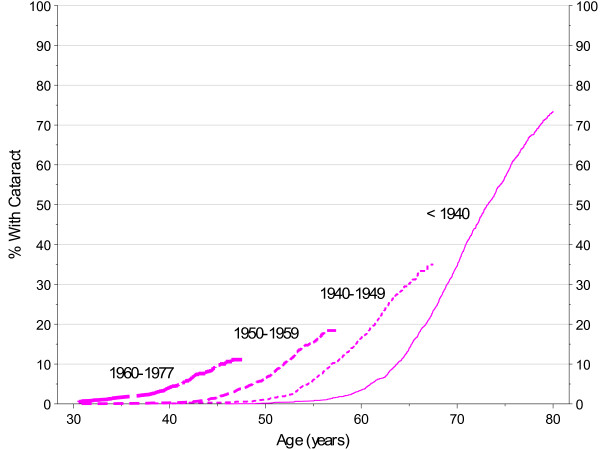
**Cataract Incidence by Age, Kaplan-Meier Estimates by Birth Cohort in Females with No Diabetes**.

Rates of exposure to potential risk factors for cataract, including such things as diet, exercise, smoking, medications, and exposure to sunlight, have changed substantially over the last century [17-22]. Given the wide age range in PMRP, it was important to consider when subjects were born when evaluating associations of risk factors with the age-specific incidence of cataract in order to avoid confounding among factors where the rate of exposure had changed over time. Compounding the need to adjust for birth year, although many clinical diagnoses are available as early as 1960 in the Marshfield Clinic electronic health record, cataract and other diagnoses from the ophthalmology department became available only much later, in the period from 1992 to 1994. Figure 5 shows cataract incidence by birth cohort in females without diabetes and shows a strong trend for earlier incidence in subjects born more recently. While some of this trend may be due to changing exposures, the greatest factor is likely the historical truncation of the EHR. At this point in time, there is little ability to detect, for example, diagnoses prior to age 50 in patients born before 1950. Largely for this reason, potential risk factors for cataract were analyzed in two different ways: 1) age at first cataract was analyzed with proportional hazards models stratified by birth cohort; and 2) 2007 prevalence of cataract was analyzed with logistic regression models. The first approach (age at first cataract) provides efficient analyses but may be particularly sensitive to historical limits on data availability. The second approach (prevalence) will be more robust to these data limitations but is not fully efficient in the use of the data (e.g., a subject age 70 having a cataract for 1 year appears the same as another subject age 70 having a cataract for 10 years).

Table 2 summarizes the results of the analyses of age at first cataract for the risk factors of interest. Model results for gender alone are included, as are results for diabetes stratified by gender. Models for the other factors of interest were fit in only those patients without diabetes and were stratified by both gender and birth cohort. The significance of each potential risk factor (Main Effect) is shown as well as a test for differences by birth cohort (Interaction).

**Table 2:** Proportional hazards model for cataract-free survival stratified by birth cohort *

Factor	Gender	Group	N	Median	# Events	Main Effect p-value	Interaction with cohort p-value	Hazard Ratio < 1940	95% Lower	95% Upper	Hazard Ratio 1960-77	95% Lower	95% Upper
Gender	Female		9305	--	2167	--	--	--	--	--	--	--	--
	Male		7031	--	1437	< .001	0.007	0.85	0.79	0.92	0.50	0.36	0.70
Diabetes	Female	No	7999	--	1567	--	--	--	--	--	--	--	--
		Yes	1306	--	600	< .001	0.128	1.26	1.13	1.41	1.80	1.02	3.18
	Male	No	5788	--	910	--	--	--	--	--	--	--	--
		Yes	1243	--	527	< .001	0.060	1.36	1.20	1.54	2.34	1.05	5.22
Smoking (ever)	Female	No	5518	--	1355	--	--	--	--	--	--	--	--
		Yes	1427	--	170	0.029	0.079	1.31	1.03	1.67	0.74	0.48	1.16
	Male	No	3061	--	520	--	--	--	--	--	--	--	--
		Yes	1255	--	113	0.632	0.287	1.07	0.81	1.43	0.68	0.33	1.38
Steroid Use	Female	No	7751	--	1581	--	--	--	--	--	--	--	--
		Yes	1554	--	586	0.043	< 001	1.12	1.00	1.26	2.26	1.50	3.39
	Male	No	5919	--	991	--	--	--	--	--	--	--	--
		Yes	1112	--	446	0.590	0.017	1.04	0.91	1.18	2.32	1.04	5.16
Statin Use	Female	No	6616	--	1086	--	--	--	--	--	--	--	--
		Yes	2689	--	1081	< .001	0.952	1.27	1.15	1.41	1.37	0.82	2.31
	Male	No	4309	--	559	--	--	--	--	--	--	--	--
		Yes	2722	--	878	< .001	0.947	1.24	1.09	1.41	1.46	0.77	2.76
BMI Category	Female	Lowest	3100	23.0	754	--	--	--	--	--	--	--	--
		Middle	3103	28.0	720	0.887	--	0.95	0.84	1.07	0.81	0.54	1.21
		Highest	3102	35.7	693	0.251	0.789	1.00	0.88	1.14	0.87	0.59	1.29
	Male	Lowest	2343	24.9	510	--	--	--	--	--	--	--	--
		Middle	2343	28.8	475	0.953	--	1.11	0.96	1.29	0.70	0.34	1.46
		Highest	2345	33.9	452	0.409	0.235	1.06	0.92	1.24	1.02	0.53	1.97
HDL Category	Female	Lowest	1409	48.5	336	--	--	--	--	--	--	--	--
		Middle	1411	58.7	328	0.707	--	1.20	0.99	1.45	1.09	0.63	1.88
		Highest	1410	70.4	293	0.965	0.795	1.02	0.83	1.25	1.24	0.72	2.13
	Male	Lowest	1166	37.7	290	--	--	--	--	--	--	--	--
		Middle	1169	46.2	240	0.145	--	0.98	0.80	1.21	0.65	0.25	1.69
		Highest	1168	55.9	218	0.731	0.553	0.92	0.74	1.14	0.98	0.42	2.25
Antioxidant Category	Female	Lowest	1959	-0.8	481	--	--	--	--	--	--	--	--
		Middle	1962	-0.0	467	0.773	--	1.07	0.91	1.25	0.82	0.49	1.37
		Highest	1961	0.7	474	0.149	0.465	1.01	0.86	1.18	0.98	0.59	1.61
	Male	Lowest	1305	-0.6	292	--	--	--	--	--	--	--	--
		Middle	1308	0.1	281	0.163	--	0.92	0.76	1.12	0.84	0.32	2.17
		Highest	1307	0.8	291	0.309	0.434	0.96	0.79	1.16	0.55	0.18	1.64

* Models for factors other than gender and diabetes were fit in subjects without diabetes.

BMI = body mass index; HDL = high density lipoprotein

Table 3 summarizes the results of the analyses of 2007 prevalence for the risk factors of interest. Model results for gender alone are included, as are results for diabetes stratified by gender. Models for the other potential risk factors were fit in only those patients without diabetes, and were stratified by gender and adjusted for age. The significance of each potential risk factor (Main Effect) is shown as well as a test for changes in the odds ratio by age (Interaction).

**Table 3:** Logistic models for prevalence of age-related cataract *

Factor	Gender	Group	N	Median	# Events	Main Effect p-value	Interaction with age p-value	Odds Ratio Age 40	95% Lower	95% Upper	Odds Ratio Age 70	95% Lower	95% Upper
Gender	Female		8401	--	1988	--	--	--	--	--	--	--	--
	Male		6028	--	1241	< .001	0.010	0.56	0.45	0.70	0.76	0.69	0.84
Diabetes	Female	No	7238	--	1470	--	--	--	--	--	--	--	--
		Yes	1163	--	518	0.036	0.272	1.92	1.31	2.83	1.55	1.33	1.81
	Male	No	4971	--	804	--	--	--	--	--	--	--	--
		Yes	1057	--	437	0.003	0.072	2.82	1.75	4.53	1.85	1.56	2.19
Smoking (ever)	Female	No	5002	--	1246	--	--	--	--	--	--	--	--
		Yes	1266	--	157	0.005	0.006	0.59	0.39	0.89	1.31	0.95	1.81
	Male	No	2650	--	469	--	--	--	--	--	--	--	--
		Yes	1039	--	91	0.014	0.032	0.40	0.20	0.76	0.95	0.64	1.40
Steroid Use	Female	No	7001	--	1475	--	--	--	--	--	--	--	--
		Yes	1400	--	513	< .001	0.002	2.44	1.75	3.42	1.38	1.15	1.66
	Male	No	5109	--	899	--	--	--	--	--	--	--	--
		Yes	919	--	342	0.014	0.040	2.35	1.31	4.22	1.31	1.05	1.64
Statin Use	Female	No	5903	--	995	--	--	--	--	--	--	--	--
		Yes	2498	--	993	0.261	0.494	1.39	0.94	2.05	1.21	1.04	1.41
	Male	No	3541	--	467	--	--	--	--	--	--	--	--
		Yes	2487	--	774	0.474	0.573	1.25	0.77	2.03	1.09	0.90	1.31
BMI Category	Female	Lowest	2407	22.71	461	--	--	--	--	--	--	--	--
		Middle	2418	27.36	571	0.670	0.563	0.92	0.66	1.27	0.88	0.73	1.06
		Highest	2413	34.50	438	0.471	0.128	0.89	0.64	1.23	0.67	0.55	0.82
	Male	Lowest	1657	24.81	286	--	--	--	--	--	--	--	--
		Middle	1657	28.38	295	0.336	0.203	0.74	0.45	1.22	1.04	0.83	1.30
		Highest	1657	32.99	223	0.844	0.702	0.68	0.41	1.14	0.74	0.58	0.93
Adjusted HDL	Female	Lowest	1234	49.19	351	--	--	--	--	--	--	--	--
		Middle	1235	59.30	224	0.593	0.637	1.14	0.72	1.81	0.93	0.72	1.21
		Highest	1235	70.91	189	0.885	0.756	1.04	0.64	1.66	0.87	0.66	1.14
	Male	Lowest	965	38.46	273	--	--	--	--	--	--	--	--
		Middle	965	46.88	136	0.594	0.754	0.81	0.42	1.56	0.77	0.58	1.03
		Highest	965	56.73	135	0.449	0.384	1.04	0.55	1.97	0.78	0.58	1.04
Antioxidant Category	Female	Lowest	1602	-0.79	374	--	--	--	--	--	--	--	--
		Middle	1603	-0.03	370	0.112	0.087	0.71	0.47	1.08	1.15	0.93	1.42
		Highest	1603	0.66	327	0.936	0.956	0.82	0.55	1.23	1.06	0.85	1.32
	Male	Lowest	1009	-0.61	208	--	--	--	--	--	--	--	--
		Middle	1009	0.12	184	0.935	0.918	1.00	0.50	1.99	1.00	0.77	1.30
		Highest	1009	0.80	171	0.857	0.825	0.96	0.48	1.92	1.03	0.79	1.36

* All models include age and interactions with age. Models for factors other than gender and diabetes were fit in subjects without diabetes.

BMI = body mass index; HDL = high density lipoprotein

**Figure 6:**
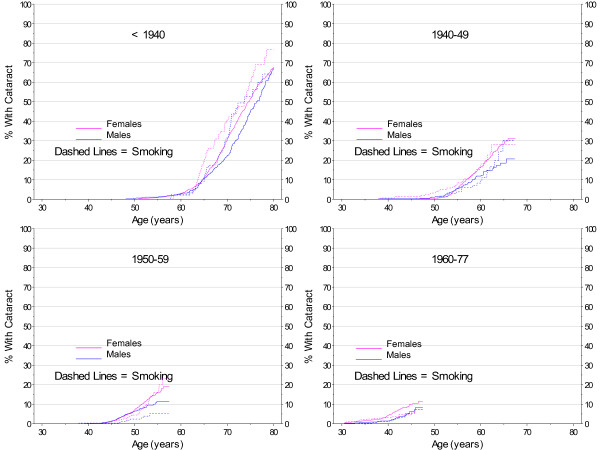
**Kaplan-Meier Estimates for Cataract Incidence by Smoking, Gender, and Birth Cohort with No Diabetes**.

**Figure 7:**
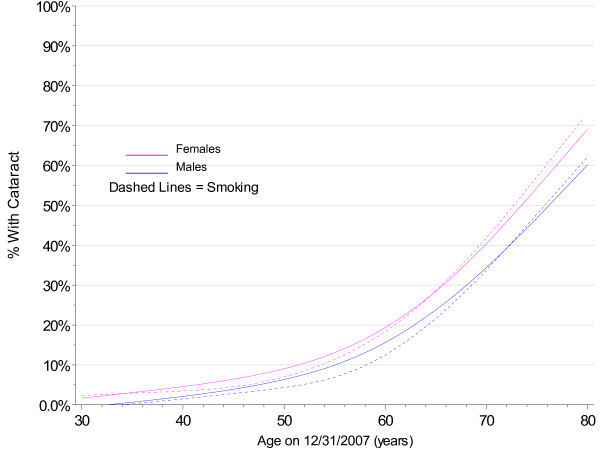
**Smoothed 2007 Prevalence of Cataract by Gender and Smoking Cohort with No Diabetes**.

Evidence of the impact of smoking on cataract development was most clear in the oldest cohort. Figure 6 displays the differences in the age cohorts. The estimate of age at cataract is earlier for the oldest smokers with a less clear distinction for each of the younger cohorts, resulting in the suggestion of a protective factor with decreased age. Figure 7 also shows the interaction of smoking and age.

**Figure 8:**
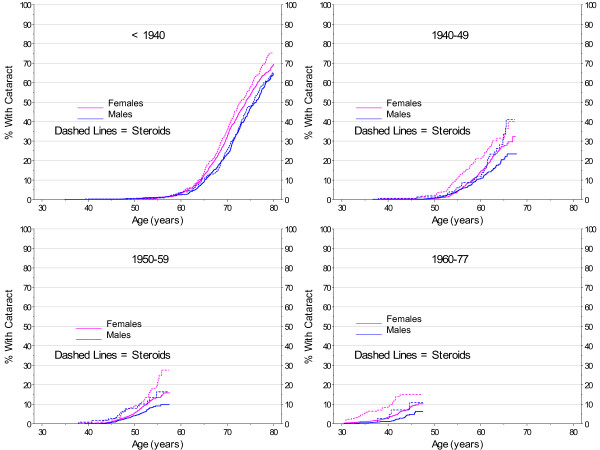
**Kaplan-Meier Estimates for Cataract Incidence by Steroid Use, Gender and Birth Cohort with No Diabetes**.

**Figure 9:**
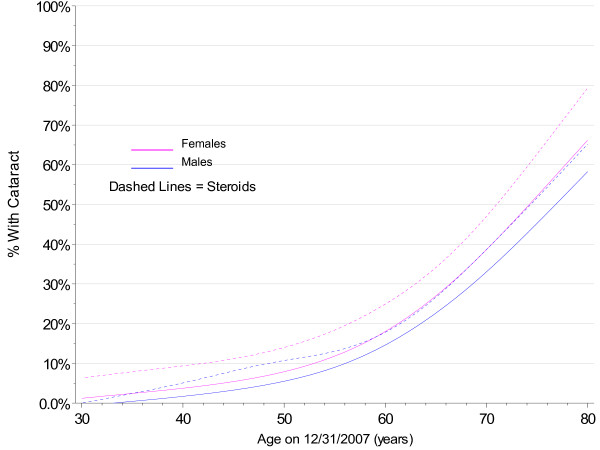
**Smoothed 2007 Prevalence of Cataract by Gender and Steroid Use Cohort with No Diabetes**.

The use of steroids gave a more consistent picture. Using steroids increases the risk of developing cataract. Shown in Figures 8 and 9, cataracts tend to develop earlier for all ages when steroids have been used. This result was apparent even without adjustment for dosage or duration of use for a steroid, only a presence or absence of selected drugs.

**Figure 10:**
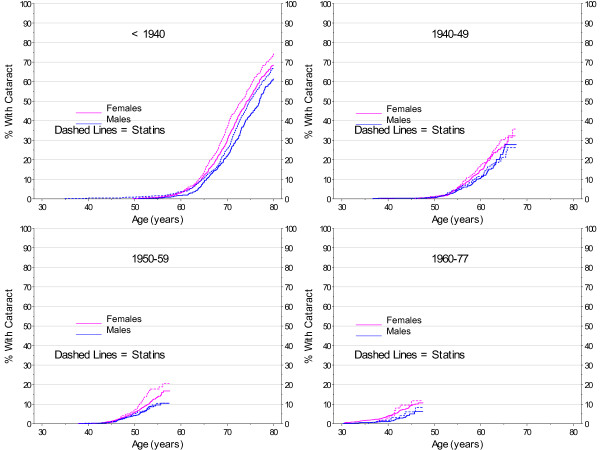
**Kaplan-Meier Estimates for Cataract Incidence by Statin Use, Gender, and Birth Cohort with No Diabetes**.

**Figure 11:**
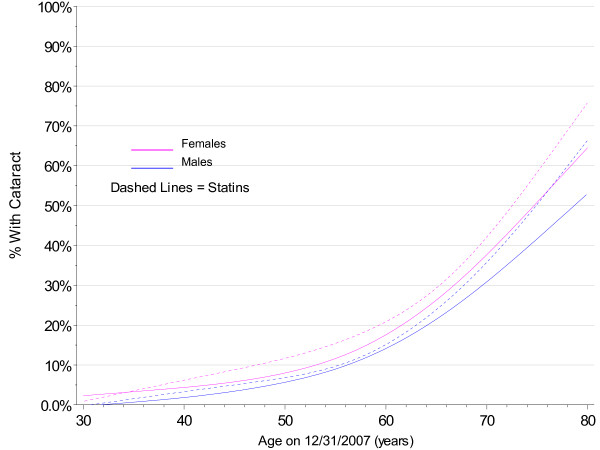
**Smoothed 2007 Prevalence of Cataract by Gender and Statin Use Cohort with No Diabetes**.

The analyses on use of statins are shown in Figures 10 and 11 and indicate a possible increase in risk for cataract development. The survival analyses (Figure 10) show significant main effects (p < 0.001) for both females and males. The hazard ratio for the earliest birth cohort was 1.27 for females and 1.24 for males using statins.

**Figure 12:**
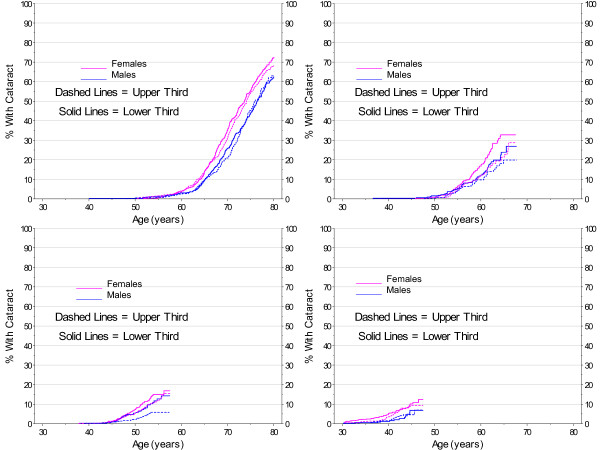
**Kaplan-Meier Estimates for Cataract Incidence by BMI (lower vs. upper third), Gender, and Birth Cohort with No Diabetes**.

**Figure 13:**
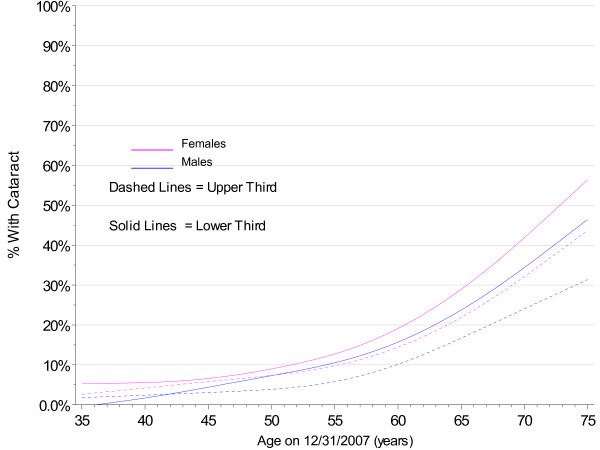
**Smoothed 2007 Prevalence of Cataract by Gender and BMI (lower vs. upper third) Cohort with No Diabetes**.

While not significant, the analyses (Figures 12 and 13) are in the direction of a protective effect with increased BMI. In the prevalence analyses, (Table 3), the odds ratio for the oldest cohort was .67 for females and .74 for males.

**Figure 14:**
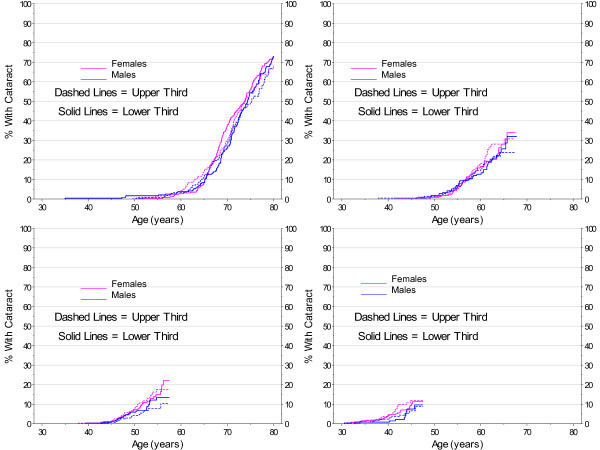
**Kaplan-Meier Estimates for Cataract Incidence by HDL (lower vs. upper third), Gender, and Birth Cohort with No Diabetes**.

**Figure 15:**
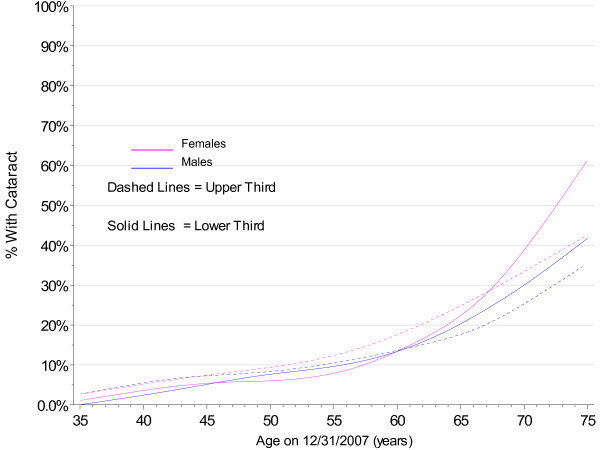
**Smoothed 2007 Prevalence of Cataract by Gender and HDL (lower vs. upper third) Cohort with No Diabetes**.

**Figure 16:**
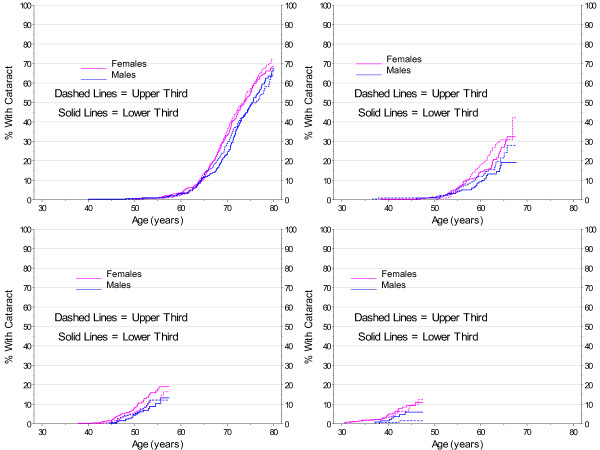
**Kaplan-Meier Estimates for Cataract by Gender and Antioxidants (lower vs. upper third) Cohort with No Diabetes**.

**Figure 17:**
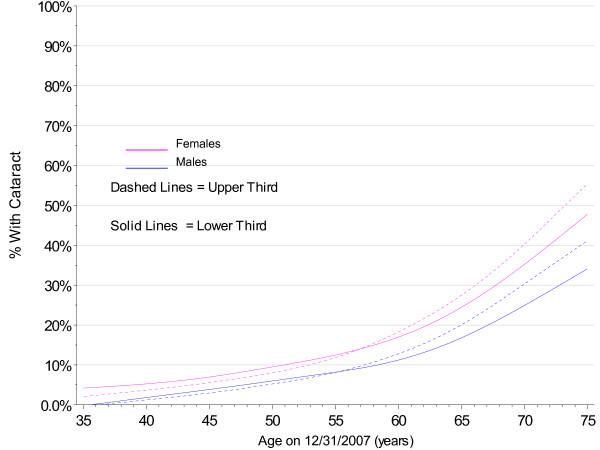
**Smoothed 2007 Prevalence of Cataract by Gender and Antioxidants (lower vs. upper third) Cohort with No Diabetes**.

Consistent with the Framingham Study [23], no clear association was found between HDL and cataract. Results shown in Figures 14 and 15 comparing those with the highest and the lowest HDL vary substantially with increasing age. Similarly, no clear findings were found for antioxidants. Shown in Figures 16 and 17, the results vary substantially with age, and do not reach statistical significance.

## Discussion

The estimates for cataract prevalence were notably higher in PMRP above age 65 compared with the EDPRG, but this may be due in part to the sensitivity of the electronic criteria in PMRP to pick up low severity cataract. However, the prevalence of surgery in PMRP is also considerably higher above age 65, suggesting population differences that might include more extensive healthcare utilization in the population- based PMRP cohort.

Being female and having diabetes were clearly associated with cataract development. This has been shown in other studies as well [24-26]. Because of this, analyses of other risk factors in the current study were limited to those without diabetes and were stratified by gender.

Some studies indicate a connection between smoking and cataract development [24-26]. Analyses in the current study were less clear. The suggestion of a possible protective effect at earlier ages could well be a limitation of the data, since younger subjects generally have less need for regular health care visits and may not be getting standard eye exams to have cataract diagnosed, or this may be due to the lack of information related to number of pack years.

As with other studies [27-29], the use of steroids was also predictive of cataract development. Odds ratios in the current study ranged from 1.31 to 2.44 for males and females across all ages, while those found by Curtis [29] ranged from 1.19 to 1.83 for cumulative dose.

Risk factors (age, female, diabetes, and steroids) that have been found to be robust or conclusive were also identified in the current study. It should be noted that the risk factors (statins, BMI, HDL, antioxidants) where results in the current study differed from other studies or were not found, have been ones that have previously had limited or conflicting results. For statins, the current study showed some increase in risk, the opposite of what has been seen in some other studies [30,31]. However, the analyses were done on ever/never use of drug with no distinction between drugs, dosage or duration, and with no adjustment for actual lipid levels. Other studies have seen a trend toward BMI as a risk factor [32-34], where the current study saw a possible trend as a protective factor. For antioxidants, the current study also found (as in previous research) that there were no consistent results related to nutrition and dietary supplements.

As cataract type could not be reliably and consistently discerned, the analyses were conducted for the presence of any cataract. The vast majority of cataract type, when indicated, were nuclear (> 96%). As prospective studies can undertake analyses based on cataract type, this may explain some of the differences found in the current study.

The differences observed in gender are potentially due to a combination of genetic factors and differences in exposure or the clinical manifestations of diabetes, but this retrospective analysis may also be confounded with differences in healthcare utilization. Women, in general, not only have recognized differences in potentially important exposures but also visit healthcare providers more frequently than do men, at least at younger ages [35]. In general, health risks due to smoking may decline after cessation, perhaps returning to near baseline after a number of years [36]. In addition, even though risks for those who recently stopped smoking are likely similar to those for current smokers, it is possible that early disease symptoms or clinical diagnoses may encourage cessation.

Exposures were recorded as available in the EHR, and in some cases (e.g., dietary intake) may reflect measures subsequent in time to cataract development. This is a recognized limitation of the electronic analysis and would introduce measurement error in analyses of risk to the degree that the exposure as recorded did not provide a good estimate of the subject's exposure prior to developing cataract.

Using EHR data has proven to be a viable tool for research. Consistent with other studies, the well documented risk factors of age, gender, diabetes and steroid use were found using an electronic algorithm to identify the presence of cataract by mining diagnosis, medication, and lab data from the EHR. This indicates that the EHR is a practical, cost effective, and an increasingly available resource for doing research. However, there are elements that need to be considered when using data mined from EHRs.

While most research studies follow their cohort over time, EHRs work with data available in clinical charts. The EHR provides a wealth of information, but there are also difficulties with doing research based on information collected from clinical treatment. For many subjects, information is available over a long period of time; however, people can move into and out of the clinical setting, resulting in minimal information or gaps in information. There may also be problems with data availability due to different departments going 'electronic' at different times. In the Marshfield Clinic system, the ophthalmology and dermatology departments were the last departments to be brought into the electronic record system because of their heavy use of drawings and diagrams. Also, there are limitations on data historically that may vary by data type (i.e., lab values were available over a longer period of time than surgery data).

Specific to this study, eye care could have been obtained at other facilities with referral into our system for surgery, well after cataracts first developed. This could delay the first diagnosis until the time surgery was needed. Research data are gleaned from data recorded by various providers in the system, which does not allow for standardized collection, grading, and documentation of the data. With the EHR, clinical data are gathered in both coded and textual format and added to the EHR at the time of the patient visit. The data are not restricted to a predefined data set or a limited data collection period. Using EHR data can be a cost effective way to determine phenotypes for use in research. While broad phenotypes can be determined using EHR, it may be less useful in determining specifics, in this case type of cataract. Missing specificity would be an argument for encouraging more specific coding to make information more useful beyond the scope of billing purposes. Developing a focus on the 'bigger picture' would open up opportunities to use collected data beyond a single intended purpose. One problem noted was a bias that developed due to the increase of frequency of eye exams for individuals diagnosed with diabetes. Because of this, cataracts were documented earlier in those with diabetes and at a higher rate due to referral into the Marshfield Clinic system and/or their more regularly scheduled ophthalmic exams.

### Strengths

Strengths of this study include being population-based with a large sample size from a stable cohort with medical records available over a long period of time. Using the EHR also allows for being able to continually add information so that data are not restricted to a limited collection period. Another strength is that age at diagnosis was able to be reliably ascertained, a common shortcoming in other studies.

### Limitations

Data were not collected under a standardized protocol, but instead were based on clinical care as recorded in the EHR. With data collected in this manner, there are variations over time (it was not uncommon for severity of cataract to 'bounce' around even with the same provider) and between treatment providers (different treatment providers may give different severity, even when seen at the same time or within in a small timeframe [referral/consultation]) in the subjective ratings of cataract severity. No distinction was made between severity of cataract or type of cataract made by opticians, optometrists, and ophthalmologists. Some subjects have limited data available as they may move in and out of the system, seek some of their care at other facilities, or come in as referrals for surgery. While not being able to determine cataract type was not a major limitation in determining the usefulness of EHR data in research, the ideal would be to have type identified. Different types of cataracts can have different risk factors, so working towards better understanding of cataract development and prevention would be enhanced by being able to determine cataract type.

## Conclusion

Using coded EHR data is a viable and efficient means to identify subjects with cataract for research, but the data for most subjects were not specific enough at our institution to identify type. The next steps will be to develop electronic algorithms and tools to better identify cataract type. It will be important to see how well these algorithms transfer to other EHR systems. Another future step will be to move towards modeling that would include genetic and other environmental factors.

## List of abbreviations

BMI: body mass index; CPT: Current Procedural Terminology; DHQ: Dietary History Questionnaire; EDPRG: Eye Diseases Prevalence Research Group; EHR: electronic health record; eMERGE: electronic MEdical Records and Genomics; HDL: high density lipoprotein; ICD-9: International Classification of Diseases, 9th revision; ICR: Intelligent Character Recognition; NLP: Natural Language Processing; PMRP: Personalized Medicine Research Project.

## Competing interests

The authors declare that they have no competing interests.

## Authors' contributions

CW completed data abstraction and prepared the initial draft of the paper. RB carried out the statistical analyses. JL developed the electronic algorithm to identify cases/controls and created the data-bases and data sets. LR configured and executed the NLP and ICR programs. PP oversaw the informatics components of the study. LC was the content expert and provided training for data abstraction. CM was Principal Investigator and designed the study and analysis plan. All authors read and approved the final manuscript.

## Pre-publication history

The pre-publication history for this paper can be accessed here:

http://www.biomedcentral.com/1471-2415/11/32/prepub
